# From Agents to Governance: Essential AI Skills for Clinicians in the Large Language Model Era

**DOI:** 10.2196/86550

**Published:** 2026-01-14

**Authors:** Weiping Cao, Qing Zhang, Jialin Liu, Siru Liu

**Affiliations:** 1 Department of Cardiology West China Hospital Sichuan University Chengdu, Sichuan China; 2 Department of Cardiology The People’s Hospital of Leshan Leshan, Sichuan China; 3 Information Center West China Hospital Sichuan University Chengdu China; 4 Department of Medical Informatics West China Medical School Sichuan University Chengdu China; 5 Department of Biomedical Informatics Vanderbilt University Medical Center Nashville, TN United States

**Keywords:** large language model, clinician, agent, competency, artificial intelligence, education, continuing medical education

## Abstract

Large language models are rapidly transitioning from pilot schemes to routine clinical practice. This creates an urgent need for clinicians to develop the necessary skills to strike the right balance between seizing opportunities and taking accountability. We propose a 3-tier competency framework to support clinicians’ evolution from cautious users to responsible stewards of artificial intelligence (AI). Tier 1 (foundational skills) defines the minimum competencies for safe use, including prompt engineering, human–AI agent interaction, security and privacy awareness, and the clinician-patient interface (transparency and consent). Tier 2 (intermediate skills) emphasizes evaluative expertise, including bias detection and mitigation, interpretation of explainability outputs, and the effective clinical integration of AI-generated workflows. Tier 3 (advanced skills) establishes leadership capabilities, mandating competencies in ethical governance (delineating accountability and liability boundaries), regulatory strategy, and model life cycle management—specifically, the ability to govern algorithmic adaptation and change protocols. Integrating this framework into continuing medical education programs and role-specific job descriptions could enhance clinicians’ ability to use AI safely and responsibly. This could standardize deployment and support safer clinical practice, with the potential to improve patient outcomes.

## Introduction

The convergence of generative artificial intelligence (AI) and health care is catalyzing a paradigm shift in clinical practice, with significant implications for the future of medicine [1-3]. Large language models (LLMs), exemplified by recent advances, such as GPT-4 and Gemini, demonstrate a transformative capacity to process multimodal data and generate context-aware responses, increasingly positioning them as integral components in frontline clinical decision support [4,5].

Although LLMs have the potential to improve clinical effectiveness, ensuring that their application optimizes patient safety, ethical alignment, and long-term benefits remains a substantial challenge [2,5]. This complexity is compounded by the intersection of regulatory requirements and ethical obligations. Evolving legal frameworks, such as the European Union (EU) AI Act and the US Food and Drug Administration (FDA) guidance, explicitly mandate human oversight for high-risk AI systems [6,7]. Simultaneously, global ethical standards from the World Health Organization (WHO) and the American Medical Association emphasize the necessity of physician leadership and accountability [2,8,9]. However, a gap remains in translating these high-level mandates into actionable clinical skills. Without the active leadership and input of clinicians, these technologies risk imposing unintended burdens and may fail to achieve their full potential [1].

The imperative for advanced AI governance arises from a fundamental shift from passive information retrieval to autonomous task execution. While conventional LLM paradigms rely on user-initiated prompt response exchanges, clinicians query the model and verify its text outputs, and the system does not autonomously call external tools. By contrast, emerging agentic workflows introduce a perceive-plan-act (and often reflect) loop [10]. In this mode, the system interprets high-level clinical intents (eg, hypertension management); decomposes them into subtasks; and autonomously executes actions via application programming interfaces, such as accessing electronic health record (EHR) data or calculating risk scores [11,12]. This transition reframes supervision; clinicians must move beyond prompt engineering to govern how autonomy is delegated, how actions are constrained, and how escalation pathways are formalized.

To address these regulatory, ethical, and technical demands, we propose a foundational, tiered AI competency framework for clinicians. The framework is structured around progressive tiers: tier 1 (foundational skills), tier 2 (intermediate skills), and tier 3 (advanced skills). We describe the core competencies at each tier, outline the framework’s limitations, and propose priority directions for validation to sustain its relevance amid an evolving regulatory landscape.

## AI Competency Framework

**Figure 1:**
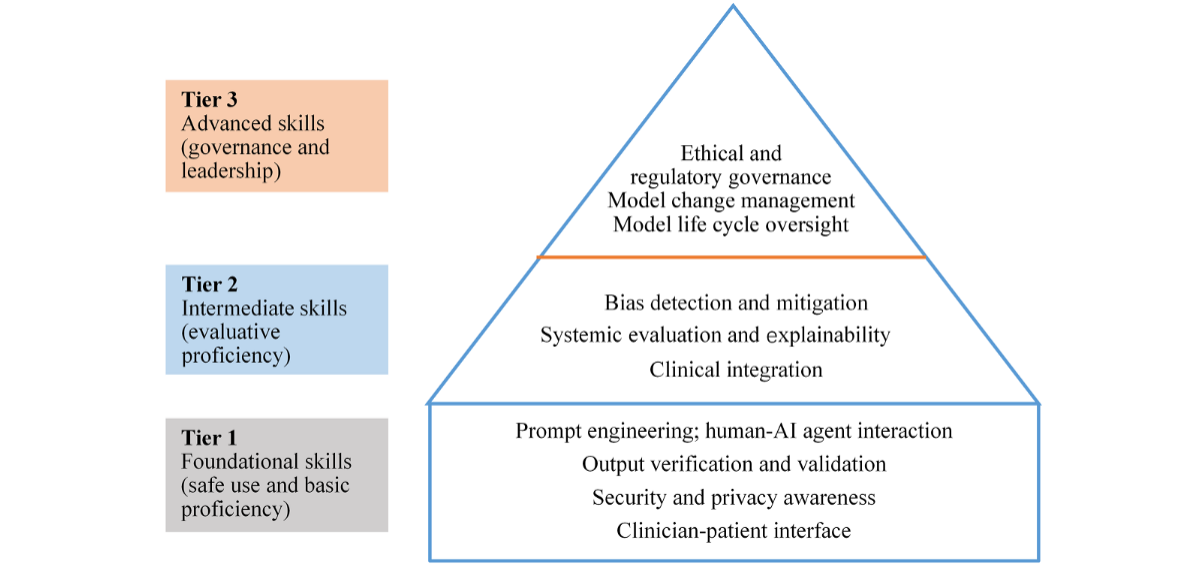
Essential artificial intelligence (AI) skills for clinicians in the large language model era.

LLM-enabled care necessitates a transition in the roles of clinicians (physicians, nurses, pharmacists, and allied health professionals)—from interpreting predictive outputs to supervising agentic workflows. Drawing on previous research, a narrative synthesis of evolving digital health competencies, and an analysis of the technical capabilities of LLMs [13-17], we propose a 3-tier, governance-aligned framework that articulates core LLM competencies. As illustrated in Figure 1, the framework progresses from foundational safe use (tier 1) to evaluative proficiency (tier 2) and ultimately to governance and leadership (tier 3).

### Tier 1: Foundational Skills (Safe Use and Basic Proficiency)

These entry-level competencies prioritize basic interaction with LLMs, enabling clinicians to leverage AI for routine tasks without compromising clinical autonomy. The key elements are described subsequently. First, prompt engineering (task specification for clinician-initiated and hybrid workflows) is used to craft precise, context-aware instructions—with explicit roles, required inputs, constraints, task steps, and output formats (including citation and traceability requirements)—to elicit task-appropriate outputs (eg, structured outlines for differential diagnosis). This competency primarily supports clinician-initiated chat and hybrid workflows, as fully agentic perceive-plan-act execution is typically governed by system-level prompts and policies rather than user-generated prompts. When paired with verification and source grounding, this approach may reduce hallucinations and improve relevance and completeness [18]. Second, human-AI agent interaction (agent supervision) ensures that agents operate within bounded autonomy with explicit roles, goals, and guardrails. Clinicians must maintain awareness of least privilege tool permissions and system constraints (eg, data minimization, time and step limits, and sandboxed execution) [19], with clear termination and escalation criteria. Clinicians also monitor and validate the perceive-plan-act-reflect loop using provenance and citation requirements; protected health information redaction; and audit logging of prompts, tool calls, and human overrides [20-22]. When confidence or calibration thresholds are not met (eg, coverage targets and abstention or deferral rules), clinicians intervene, interrupt the agent, or revert to manual workflows and document the event for review. Third, output verification and validation involve clinicians critically evaluating individual LLM outputs for accuracy, relevance, and internal consistency. Generated content (eg, summaries, diagnostic considerations, and treatment plans) is cross-referenced against the EHR, structured data, and established clinical evidence to detect hallucinations, omissions, or misstatements. In culturally diverse settings, clinicians must assess outputs for cultural safety and linguistic accuracy. This involves verifying that translated instructions and culturally specific dietary or lifestyle advice are appropriate for the patient’s context. Clinicians should also check for Anglocentric bias that could conflict with local norms or the patient’s language proficiency. This human-in-the-loop verification is essential for ensuring patient safety in individual clinical encounters [23,24]. The fourth element is security and privacy awareness. To comply with regulations such as the Health Insurance Portability and Accountability Act (HIPAA) and the General Data Protection Regulation, clinicians must adhere to foundational safeguards centered on data minimization and appropriate tool use. For nonintegrated or open access LLM interfaces, this includes avoiding entry of protected health information and direct identifiers and, when clinically necessary to discuss a case, removing or generalizing nonessential identifiers before input [2,5,25,26]. In contrast, for authorized, integrated enterprise agents operating within a secure EHR environment, manual deidentification is often neither feasible nor necessary; instead, clinicians verify minimum necessary access, confirm the agent is scoped to the correct patient context, and ensure permissions are aligned with the specific clinical task through role-based access control and least privilege settings. Rather than conducting technical audits themselves, clinicians prevent inadvertent privacy breaches by distinguishing approved tools from unapproved ones and escalating permission or access-scope concerns through institutional channels. These baseline competencies are prerequisites for safe AI use in routine clinical workflows [5]. Fifth, clinician-patient interface (transparent communication and shared decision-making) involves incorporating AI-assisted content into the clinical encounter without undermining patient trust or the therapeutic alliance. Clinicians should disclose when AI is used (eg, AI-scribed summaries, patient-portal messages, and patient education materials) to uphold patient autonomy and informed consent [26].

### Tier 2: Intermediate Skills (Evaluative Proficiency)

Building on foundational knowledge, these competencies center on critically assessing and integrating LLMs into clinical reasoning workflows while addressing bias and uncertainty in generative AI. First, bias detection and mitigation require clinicians to interpret algorithmic bias audit reports and uncertainty quantification outputs (eg, confidence intervals, prediction intervals, or conformal prediction sets when provided) to assess reliability across patient subgroups. Clinicians initiate and validate remediation actions—such as recommending prompt or workflow adjustments and defining escalation and deferral rules—in coordination with technical teams, ensuring adherence to prespecified fairness metrics and minimum subgroup performance thresholds [27,28]. For example, in tumor grading, clinicians review reported subgroup performance using minimum sample-size thresholds, calibration and coverage, abstention rates, and uncertainty displays (including confidence sets). They assess model rationale and interpret between-group performance gaps. Second, systemic evaluation and explainability involve moving beyond checking individual outputs to assessing the broader reliability, calibration, and failure modes of the AI system. Clinicians should be able to interpret model performance metrics (eg, sensitivity, specificity, error and hallucination rates, and performance in specific subpopulations) and evaluate available explainability outputs (eg, feature importance scores, reason codes, or saliency maps where available) to understand why a model reached a conclusion [29]. This evaluation must include equity audits that assess model performance across distinct subgroups (eg, race, ethnicity, and language) [28]. For instance, a model may demonstrate high overall accuracy but fail disproportionately for languages spoken by minority groups or specific dialects. Clinicians leading the evaluation must identify such disparities and determine if the model is safe for deployment in diverse populations. These skills enable clinicians to judge systemic trustworthiness and identify appropriate clinical use cases and target populations for which the model is calibrated, effective, and equitable [30]. Third, clinical integration requires clinicians to use domain knowledge to refine model outputs (eg, align treatment suggestions with evidence-based guidelines) while monitoring for potential deskilling. Clinicians maintain human-AI collaboration and specify deferral and escalation rules (eg, abstention thresholds and human-review triggers) and document these events for auditability [31,32].

### Tier 3: Advanced Skills (Governance and Leadership)

Unlike the foundational skills in tiers 1 and 2, this tier represents a specialized track for clinician-leaders, clinical informaticists, and physician builders assuming governance roles. These competencies focus on the strategic oversight and architectural direction of AI systems. The main competencies involved are described subsequently. First, ethical and regulatory governance involves overseeing the development of institutional policies for LLM use to ensure alignment with ethical principles, data protection laws (eg, General Data Protection Regulation and HIPAA), and international guidance [26]. This requires establishing governance infrastructure—such as AI steering committees and ethics review boards—to specify authorized use cases, roles and responsibilities, liability frameworks, and compliance protocols. Crucially, policies must explicitly delineate accountability boundaries among supervising clinicians, health care institutions, and AI developers and vendors, particularly for autonomous or semiautonomous agentic workflows. In this capacity, clinician leaders do not personally conduct technical audits; instead, they serve as the strategic link between medical staff and technical bodies, ensuring that institutional processes reflect clinical realities and patient safety risks. Second, model change management requires supervision of domain adaptation (eg, task- or specialty-specific tuning) within a multidisciplinary process. In this capacity, clinicians bridge clinical needs and technical implementation, upholding standards for validity, equity, and safety. This supervision necessitates predefined evaluation plans, comprehensive documentation (eg, model cards), and rigorous external validation (including multicenter, temporal, and geographic shift tests) before production deployment. Leaders must specify minimum performance thresholds and mandate shadow deployment phases to validate safety before full patient exposure [33,34]. Third, model life cycle oversight entails governing AI systems across their full life cycle—from validation through postmarket monitoring, updating, and decommissioning. This includes orchestrating institutional processes for drift detection, performance re-evaluation, and version control [35,36] (Textbox 1). Leaders must navigate complex regulatory mechanisms for iterative improvement, such as the predetermined change control plans (PCCPs) by the FDA [6] and the postmarket surveillance requirements of the EU AI Act [7]. They collaborate with informatics, regulatory, and quality teams to ensure that updates, retraining, or expanded indications are clinically justified, transparently communicated, and supported by robust evidence and incident review protocols [35,37].

**Clinical vignette—governance in action.**

**Scenario: executing a manual rollback protocol**

- A clinical informatics director oversees a deployed discharge-summary agent. During routine postmarket surveillance, the monitoring dashboard signals that the model’s summarization accuracy has dropped below the prevalidated threshold of 95% (a metric specified in the Food and Drug Administration–accepted predetermined change control plan). Attributing the decline to data drift caused by a recent update in the hospital’s note-template format, the director initiates a rollback protocol—leveraging either institutional version control or a vendor-mediated pathway specified in the service-level agreement. The deployment is rolled back to the previous stable version (version 2.0) while the technical team remediates and revalidates the updated model (version 2.1).
- In deployments where direct rollback is technically unsupported (eg, some software as a service–based integrations), the protocol mandates pausing or disabling the agent and reverting to manual workflows until remediation is complete.

## Alignment and Differentiation From Existing Frameworks

This framework is broadly aligned with the American Medical Association’s guidance on augmented intelligence, prioritizing physician leadership, transparency, and patient benefit [38,39]. Furthermore, it adheres to competency-based digital education frameworks from the WHO and the Association of American Medical Colleges, both of which prioritize observable behaviors and measurable learning outcomes [40-42]. It also builds on recent competency proposals in AI and digital health that foreground digital health literacy, awareness of data bias, and the ethical use of assistive tools [10,43]. As summarized in Table 1, our contribution lies in extending these earlier frameworks to the agentic LLM era. First, we explicitly differentiate between predictive and informational paradigms and agentic workflows. Accordingly, we move from clinicians interpreting decision support outputs to supervising and governing active, tool-using agents. Second, we introduce model life cycle literacy as an explicit competency domain, encompassing familiarity with mechanisms for ongoing monitoring, updating, and regulatory adaptation. Within this broader, jurisdiction-agnostic concept, PCCPs in the FDA context are presented as one concrete example, alongside emerging requirements under frameworks, such as the EU AI Act. To our knowledge, previous frameworks have not explicitly integrated agent supervision and life cycle–oriented governance into a tiered, clinician-facing competency model.

**Table 1:** Comparison of the agent to the governance framework and existing digital health competency frameworks.

Feature and dimension	Agent to governance framework	Existing frameworks (eg, World Health Organization, American Medical Association, and Association of American Medical Colleges)
Technological scope	Agentic and autonomous: agentic workflows (perceive-plan-act loops) and tool-using large language models that execute multistep tasks	Predictive and informational: clinical decision support, diagnostic classifiers, and standard information retrieval systems
Clinician’s role	Supervisor and governor: human-on-the-loop oversight for task delegation, monitoring agent behavior, and managing bounded autonomy	Interpreter and decision-maker: human-in-the-loop integration, focusing on the critical appraisal of risk scores and diagnostic suggestions
Verification skills	Output verification and logic checking: detection of hallucinations in generative text and verification of agentic tool calls (eg, application programming interface actions)	Statistical and evidence-based appraisal: evaluation of model performance metrics (eg, sensitivity and specificity), data quality, and automation bias
Regulatory and life cycle	Life cycle management: specific literacy in predetermined change control plans, algorithmic drift detection, and postmarket surveillance (eg, European Union Artificial Intelligence Act and Food and Drug Administration)	Foundational ethics and compliance: adherence to core bioethical principles (beneficence and equity), privacy standards (Health Insurance Portability and Accountability Act and General Data Protection Regulation), and informed consent
Target audience and structure	Tiered differentiation: distinguishes between frontline users (tiers 1 and 2) and a specialized leadership track (tier 3) for governance	Universal digital literacy: baseline digital health competencies applicable to the broad health care workforce to ensure safe general use

## Operationalizing Competencies for Education and Assessment

Translation of this framework into continuing medical education (CME) curricula requires the specification of observable, assessable behaviors aligned with competency-based medical education principles. Given that the clinical workforce encompasses diverse roles—including physicians, nurses, and allied health professionals—implementation and assessment should be tailored to role-specific scope of practice and role-based EHR access controls. For example, behavioral indicators involving least privilege enforcement or the rejection of agent actions may be operationalized differently depending on the individual’s credentialed permissions and administrative privileges. To ensure implementation feasibility and mitigate workforce burden, only tier 1 competencies are intended for the general clinical workforce, whereas tiers 2 and 3 are reserved for smaller groups of superusers and clinician leaders in formal governance roles. To avoid adding entirely new courses, these competencies are designed to be integrated into existing curricula (eg, evidence-based medicine, clinical reasoning, and quality and safety) and CME activities. Institutions are responsible for resourcing and coordinating these training activities, ensuring that individual clinicians are not expected to acquire advanced competencies (tiers 2 and 3) without appropriate organizational support and protected time.

Table 2 links each tier to behavioral indicators written as active, measurable learning outcomes. Indicators span tier 1 (eg, identifying hallucinations) to tier 3 (eg, initiating life cycle protocols) and should be tailored to role-specific responsibilities and decision rights. These indicators provide curriculum developers with a concrete scaffolding to design simulation-based, workplace-based, and microlearning assessments that verify skill acquisition in clinical practice. Ultimately, these anchors facilitate the incorporation of this framework into CME curricula and clinical job descriptions, thereby promoting institutional transparency, accountability, and regulatory alignment [40,43,44].

**Table 2:** Sample behavioral indicators for continuing medical education assessment and clinical application.

Core competency		Behavioral indicator (observable action)
**Tier 1: foundational (frontline user)**		
	Prompt engineering	Formulates a context-aware prompt that includes explicit role definitions (eg, act as a cardiologist), constraints, and required output formats, without disclosing PHI^a^
	Human-AI^b^ agent interaction	Identifies and intercepts inappropriate agent requests (eg, social history for refills) and enforces denial or escalation protocols based on least privilege guardrails and predefined termination and handoff criteria
	Output verification and validation	Detects and corrects a hallucinated reference or dosage in a large language model–generated draft by cross-referencing with the patient’s structured laboratory data and trusted guidelines
	Security and privacy awareness	Uses minimum necessary data; deidentifies data for nonintegrated tools; for electronic health record agents, verifies patient context and least privilege access, and escalates PHI or policy risks
	Clinician-patient interface	Informs patients when AI is used, explains AI-derived insights in patient-appropriate language (including uncertainties and limitations), and documents consent or refusal when clinically indicated.
**Tier 2: intermediate (superuser or champion)**		
	Bias detection and mitigation	Interprets stratified subgroup performance and uncertainty reports, flags clinically meaningful disparities, triggers mitigation (eg, threshold adjustments or human-review rules), and verifies improvement via updated audit reports
	Systemic evaluation	Evaluates a confusion matrix for a diagnostic AI tool to determine if the false-negative rate is acceptable for a specific screening population
	Explainability	Interprets available explainability outputs (eg, feature importance, reason codes, or saliency maps where available) to detect spurious cues and document potential failure modes
	Clinical integration	Defines where AI outputs enter the workflow; assigns roles, documentation, and escalation steps; and maintains clinician accountability when AI recommendations conflict
**Tier 3: advanced (governance leader)**		
	Ethical and regulatory governance	Drafts and implements an institutional policy that establishes escalation pathways and explicitly delineates accountability and liability boundaries among the supervising clinician, the institution, and the AI developer for autonomous agentic workflows
	Model change management	Initiates and justifies model change requests (eg, recalibration, retraining, or expanded indication), defining the clinical rationale, validation plan, and monitoring criteria consistent with the predetermined change control plan
	Model life cycle oversight	Oversees monitoring of model performance and drift (eg, calibration, error rates, and data shifts); ensures execution of incident protocols and predefined controls (eg, roll back and human review)

^a^PHI: protected health information.

^b^AI: artificial intelligence.

## Limitations and Future Work

We propose a governance-aligned competency framework designed to guide clinicians in the safe and effective use of LLMs in clinical practice. However, several limitations should be acknowledged. First, external validity may differ by specialty, care setting (inpatient vs ambulatory), health-system maturity, and EHR integration capacity. Critically, the institutional infrastructure required for tier 3—specifically, the establishment of AI steering committees—may currently be feasible only in resource-rich academic medical centers. Mandating such governance structures in resource-constrained community hospitals may be impractical. This feasibility gap extends to global health contexts; the framework requires adaptation in low- and middle-income settings where informatics infrastructure, governance capacity, and regulatory regimes differ substantially. Moreover, the objective structured clinical examination blueprint [44] and key performance indicators remain surrogate end points. By themselves, these measures do not guarantee improvements in patient-centered outcomes (eg, adverse events and readmissions). Second, a prospective, multicenter external evaluation is still necessary. Although we specify fairness analyses and minimum subgroup sample size and performance thresholds, real-world coverage across languages, cultural contexts, pediatrics, geriatrics, and rare-disease pathways is likely incomplete. Third, the regulatory environment remains dynamic as harmonized standards under the EU AI Act and FDA change control frameworks (eg, PCCPs) continue to evolve. Accordingly, operationalized procedures and performance thresholds should be periodically reassessed—particularly following material model updates—to sustain regulatory compliance and clinical relevance. Fourth, the automation paradox (skill decay) warrants attention. While AI agents improve efficiency, they may precipitate clinician deskilling over time. Safe fallback protocols (eg, reverting to manual workflows during system failure) are feasible only if clinicians maintain underlying diagnostic and procedural competence. To mitigate this risk, organizations should integrate automation-off scenarios into simulation training and downtime contingency plans to ensure clinicians remain capable of detecting errors and safely resuming control.

Given the rapid evolution of clinical AI, this framework requires ongoing refinement across 5 strategic areas. First, validation studies use expert consensus (eg, Delphi methods) and multicenter educational trials to link tiered competencies to observable clinical behaviors, such as error interception, safe deferral, and workflow efficiency. Second, assessment science strengthens psychometric measurement by refining objective structured clinical examination stations, bias and calibration checklists, and reliability targets (eg, generalizability coefficient [G] ≥0.70) [45,46]. Third, capacity building establishes faculty development programs and reusable educational resources—including deidentified sandboxes, annotated audit log exemplars, and HIPAA-aligned exercise sets—to support cross-specialty implementation. Fourth, advanced equity and uncertainty quantification cultivates practical competence in algorithmic fairness and uncertainty management through routine subgroup audits, coverage targets and reporting (using conformal prediction where appropriate), and bedside remediation playbooks. Fifth, simulation and institutional integration evaluate the feasibility and effectiveness of embedding automation-on and automation-off scenarios and rollback procedures within existing simulation programs and downtime contingency planning (eg, multidisciplinary team discussions). Outcomes include competency attainment, error interception during failures, auditability, and pathways for formal recognition within CME credit structures and institutional job descriptions.

## Conclusions

The progressive competency model integrates technical proficiency with ethical governance to provide clinicians with essential AI skills for the LLM era. By embedding these competencies into CME standards and job descriptions—using clear, observable behaviors—institutions can standardize safe and accountable AI use. Preparing for an AI-augmented future requires integrating governance-focused skills into medical training and professional development. This approach positions clinicians as responsible stewards of AI, ensuring adoption remains sustainable and centered on patient care.

## Abbreviations

AI: artificial intelligence
CME: continuing medical education
EHR: electronic health record
EU: European Union
FDA: Food and Drug Administration
HIPAA: Health Insurance Portability and Accountability Act
LLM: large language model
PCCP: predetermined change control plan
WHO: World Health Organization

## Data Availability

Data sharing is not applicable to this paper as no new datasets were generated or analyzed during this study.
